# Case report: Ileocecal preservation for multiple small intestinal duplications

**DOI:** 10.3389/fped.2023.1205155

**Published:** 2023-06-05

**Authors:** Hongxiu Xu, Weiqiang Liu, Chunqing Liu, Yunpeng Zhai, Huashan Zhao, Rui Guo, Longfei Lv, Shisong Zhang

**Affiliations:** ^1^ Department of Thoracic and Oncological Surgery, Children’s Hospital Affiliated to Shandong University, Jinan, China; ^2^ Department of Thoracic and Oncological Surgery, Jinan Children’s Hospital, Jinan, China; ^3^ Department of Pediatric Surgery, Zhucheng Maternal and Child Health Hospital, Weifang, China; ^4^ Zhucheng Longcheng Hospital of Traditional Chinese Medicine, Weifang, China

**Keywords:** children, multiple, intestinal duplication, ileocecal junction preservation, laparoscopy

## Abstract

Small-intestinal duplication is a rare congenital developmental anomaly that is mainly single; multiple small-intestinal duplications are rare. Most malformations are located in the ileocecal region. The primary surgical treatment is complete resection of the malformations and adjacent intestinal ducts. However, the ileocecal junction plays an important role in children, and it is difficult to preserve it; multiple intestinal repairs increase the risk of postoperative intestinal fistula, which is a challenge for pediatric surgeons. Herein, we report a case of ileocecal preservation surgery for the treatment of multiple small intestinal duplication malformations near the ileocecal area. The child underwent laparoscopically assisted cyst excision and multiple intestinal repairs and had good postoperative recovery and follow-up.

## 1. Introduction

Small intestinal duplication is a rare congenital malformation with an incidence of approximately 1/4,500, and multiple small intestinal duplications are even rarer (1–3). Multiple small intestinal duplications are easily misdiagnosed because lesions can occur in any part of the small intestine. Therefore, a combined abdominal ultrasound and computed tomography (CT) examination before surgery is necessary, and laparoscopic exploration during surgery can further reduce the risk of missed diagnoses.

Surgery is the only effective treatment for small intestinal duplications. The preferred treatment is resection of the duplication together with the adjacent normal intestine, followed by primary anastomosis (3, 4). The risk of intestinal fistula after multiple intestinal resections and intestinal anastomoses is relatively higher than that after a single intestinal anastomosis (5). Deformities often occur in the ileocecal region, which plays an important role in children. Therefore, ileocecal preservation surgery is a new challenge in the treatment of multiple small intestinal duplications near the ileocecal region. Herein, we share our experience with the diagnosis and treatment of a rare case of multiple small intestinal duplications near the ileocecal region.

## 2. Case presentation

**Figure 1:**
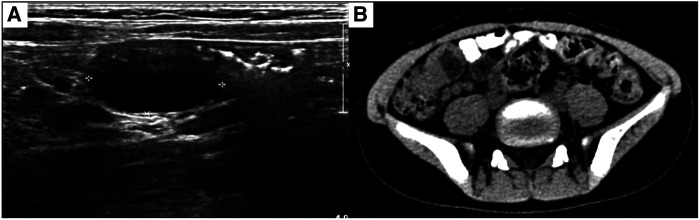
(**A**) Ultrasound revealing a cystic mass measuring 20 × 17 × 12 mm at the end of the ileum wall. (**B**) Abdominal CT showing two round cystic low-density shadows in the right lower abdomen, with sizes of 25.9 × 22.5 mm and 19.1 × 14.1 mm, respectively. The lesions are close to the bowel and show compression. ▴: Lesion in ileocecal junction; ▾: Lesion 5 cm from ileocecal junction.

A female aged 4 years and 10 months was admitted to our hospital due to paroxysmal abdominal pain for more than 2 months. The usual abdominal pain was mild, short, irregular, and spontaneously resolved. There were no symptoms of nausea, vomiting, abdominal distension, hematochezia, or other discomforts. The child was treated for gastroenteritis in a local hospital, however, her symptoms did not improve, and her parents came to our hospital for further treatment. Ultrasound examination showed a cystic mass of about 20 × 17 × 12 mm at the end of the ileum wall, which was anechoic and well transmitted. The intestinal contents passed smoothly, and a diagnosis of intestinal duplication of the terminal ileum was made. Meanwhile, abdominal CT examination showed two round cystic low-density shadows in the right lower abdomen, with sizes of 25.9 × 22.5 mm and 19.1 × 14.1 mm, respectively. The lesions were located close to the intestines and showed signs of compression (Figure 1). The patient was admitted to our hospital for surgery. The diagnosis of a small intestinal duplication was certain; however, further exploration was needed during surgery to confirm whether there was a single or multiple small intestinal duplications.

**Figure 2:**
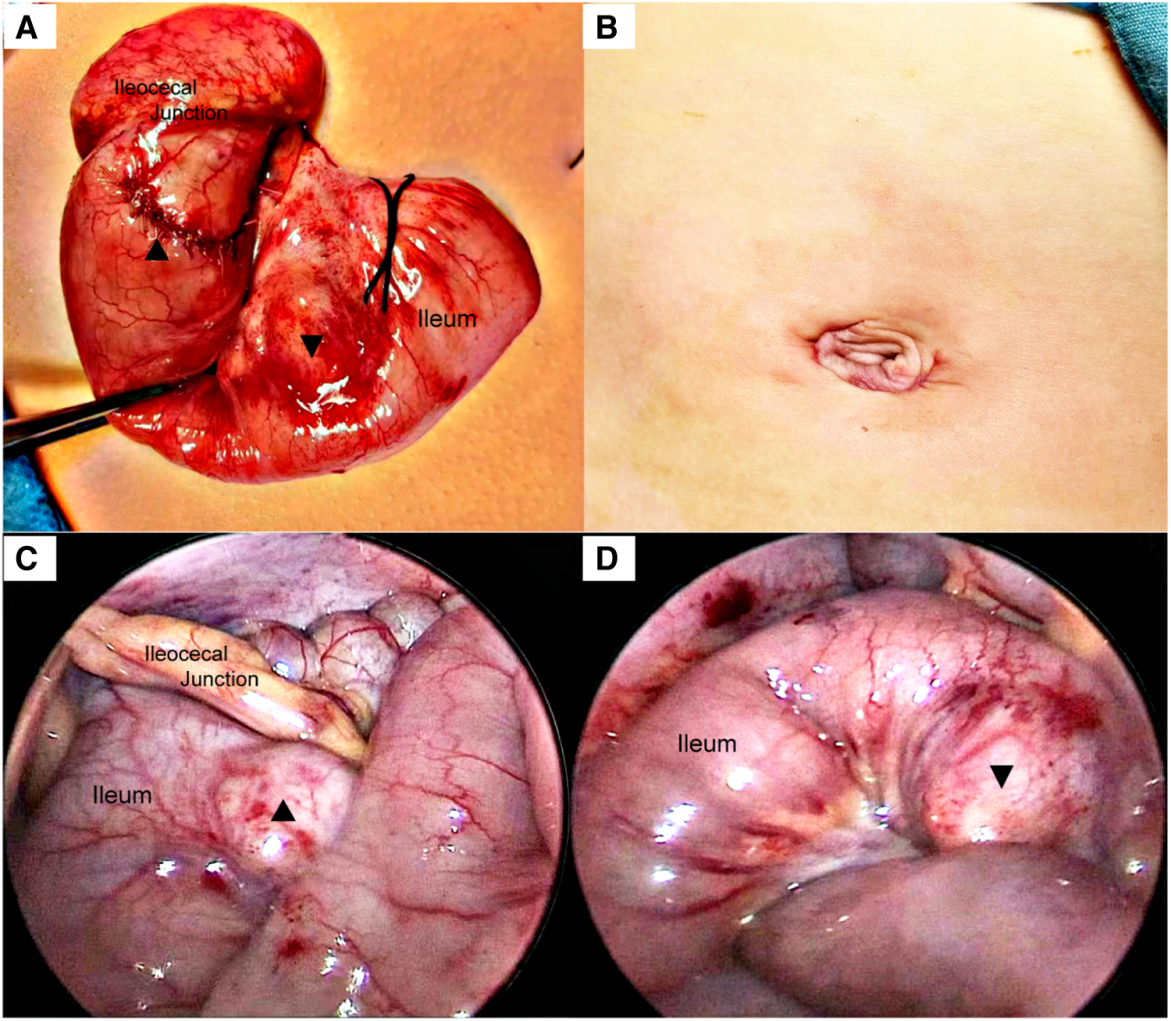
(**A**) The intestines were pulled out through the umbilicus. And the cyst mucosa was removed. (**B**) An umbilical incision along the natural skin lines reduces the local trauma and has improved esthetical outcomes. (**C**) A cystic mass, approximately 25 × 20 × 20 mm in size, immediately adjacent to the ileocecal junction found in the laparoscopic field of view. (**D**) Another cyst 5 cm from the ileocecal junction, measuring approximately 20 × 20 × 15 mm. ▴: Lesion in ileocecal junction; ▾: Lesion 5 cm from ileocecal junction.

**Figure 3:**
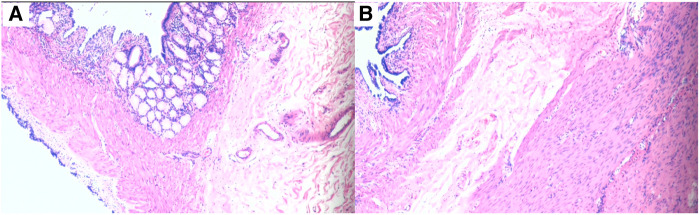
(**A)** The cyst is lined with the gastric epithelium. (**B**) The cyst is lined with the mucosal epithelium of the small intestine.

**Figure 4:**
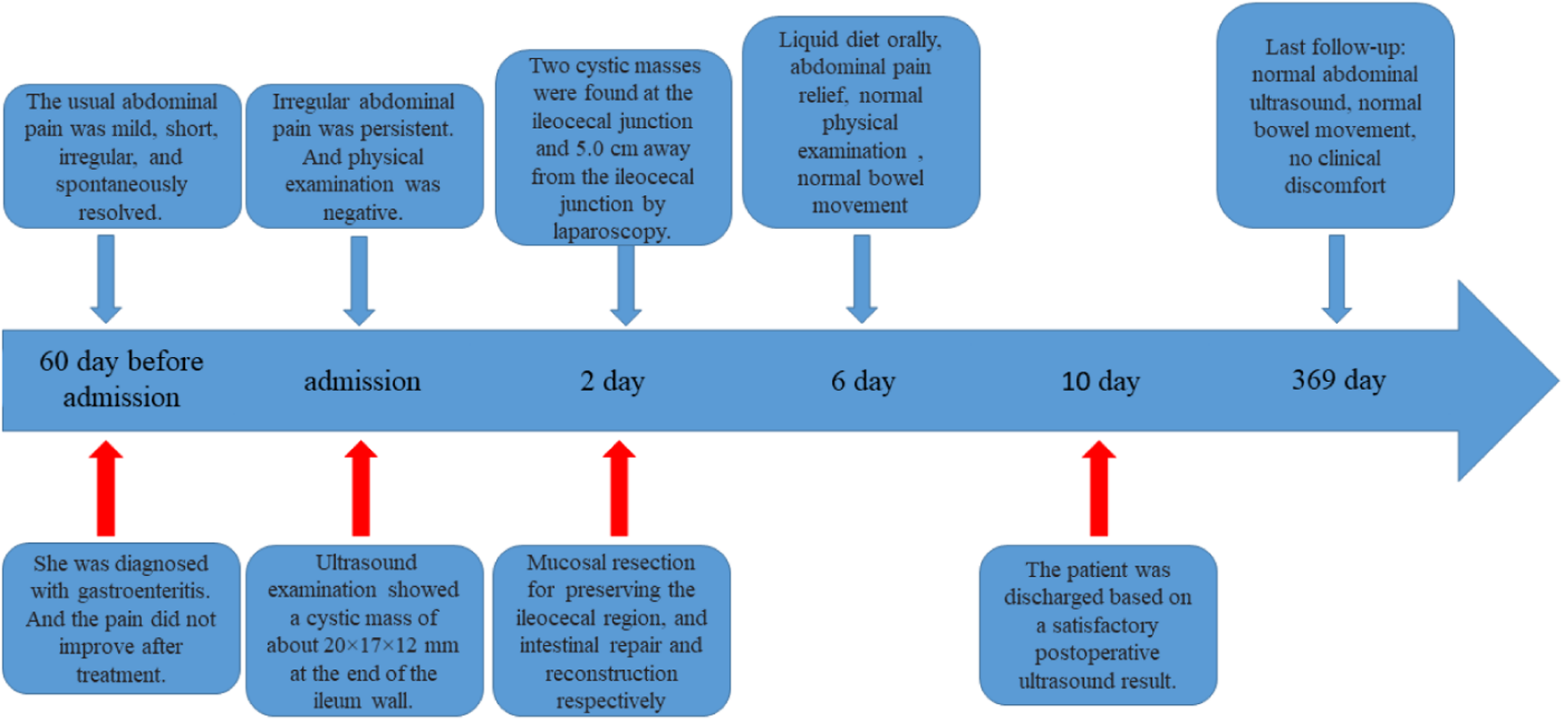
Timeline of this case.

A 5 mm incision was made at the left and right edges of the umbilicus and a 5 mm trocar was inserted. After the cyst was found, incisions at the left and right edges of the umbilicus were curved along the lower edge of the umbilicus. And the intestinal tube was removed from the umbilical incision. All intestinal tubes were examined intraoperatively by laparoscopy. Two cystic masses, approximately 25 × 20 × 20 mm and 20 × 20 × 15 mm in size, were found at the ileocecal junction and 5.0 cm away from the ileocecal junction (Figure 2), located at the mesenteric border and co-walled with the ileum, with low tension and unobstructed passage of intestinal contents. Due to the proximity of the two cysts to the ileocecal region, mucosal resection was performed to preserve the ileocecal region. The intestinal mucosa was damaged from the cysts' location within the intestinal walls, and intestinal repair and reconstruction were performed. There were no postoperative complications, such as bleeding, intestinal fistula, or intestinal stenosis, and the abdominal pain was completely relieved. Postoperative pathology confirmed cystic intestinal duplications (One was lined with ectopic gastric mucosa. And the other was lined with small intestinal mucosa) (Figure 3). The patient was able to eat orally on the 4th day after surgery and was discharged successfully on the 9th day. The patient was followed up for 1 year and recovered well. We use a timeline to show the treatment process more intuitively (Figure 4).

## 3. Discussion

There are few reports of multiple alimentary tract duplications. In the existing literatures, intestinal duplications are mostly reported to be combined with Meckel's diverticulum, gastroesophageal duplications, or intestinal malrotation. And most of them are accidentally found during imaging examinations due to clinical symptoms such as abdominal pain, ileus, intussusception, hematochezia, and dyspnea etc. Simple multiple intestinal duplications are rarely reported separately (4, 6, 7). The incidence of multiple small intestinal duplications in intestinal duplication is approximately 5% and extremely rare (6, 8). The clinical symptoms depend on the size and location of the deformity. The main manifestations are abdominal pain, abdominal mass, and obstruction symptoms caused by adjacent intestinal compression. Some malformations without clinical symptoms are incidentally observed during the imaging examinations. Due to the lack of specific clinical manifestations, small intestinal duplications are easily missed and misdiagnosed before surgery. This patient only had atypical abdominal pain, and repeated treatment for more than 2 months did not clarify the cause. Abdominal ultrasonography revealed duplication of the small intestine.

Ultrasonography is of great value for the initial diagnosis of small intestinal duplication. Xiang L et al. reported no significant difference in the diagnostic rate of small intestinal duplication between ultrasound and CT (9). However, Martini et al. believed that although ultrasound is of great value in the initial diagnosis of small intestinal duplication, CT may still provide clinical clues when it does not detect abnormalities (10). In this case, a hypoechoic mass was found in the mesentery on preoperative ultrasonography; however, on subsequent abdominal CT, we found two abnormal cystic masses in the right lower quadrant of the abdomen. Therefore, the ultrasound examination was not accurate in this case because it was somewhat subjective and could not provide clinicians with a complete image of the abdomen. CT examination compensates for the shortcomings of ultrasound. Objective CT images are easy for clinicians to read and analyze in combination with the clinical conditions. We believe that multiple small intestinal duplications are easily misdiagnosed, because lesions can occur in any part of the small intestine. Therefore, a combined ultrasound and abdominal CT examination before surgery is necessary. Moreover, laparoscopic exploration during surgery can reduce the risk of missed diagnoses. During the operation, a cyst malformation was found in the wall of the distal ileum that did not communicate with the intestinal lumen. We performed mucosal stripping of the intestinal duplications while preserving the integrity of the native intestinal structure, which is beneficial for the protection of intestinal function. We used transumbilical laparoscopic surgery because it has the following advantages: incision along the natural umbilical skin lines, improved esthetical outcomes, less trauma, faster recovery, and easy acceptance by parents. The magnifying function of laparoscopy is conducive to intraoperative observation, and a clear field of vision can reduce missed or misdiagnoses (11).

Eighty percent of intestinal duplications are cystic and most are located near the ileocecal junction (1, 3, 9). Surgery is the only effective treatment option. Because the cyst malformation shares a wall with the normal bowel and has a common blood supply, the preferred treatment is resection of the duplication and its adjacent normal bowel, followed by primary anastomosis. Ileocecal resection is typically performed for intestinal duplications near the ileocecal junction (3). However, an intact ileocecal region plays an important role in the survival and nutrition of children. Ileocecal resection can cause shortened transit time in the small intestine, impaired absorption, long-term chronic diarrhea, malnutrition, electrolyte disturbance, colonic reflux, and intestinal flora imbalance (12–15). Therefore, when multiple repetitive malformations are located near the ileocecal region and the distance is small, the difficulty of preserving the ileocecal region is further increased. Tiryaki et al. pointed out that mucosal resection alone is feasible due to special location or extensive accumulation of deformities (16, 17). At the same time, Deguchi, Catalano et al. successfully resected intestinal duplications at the end of the ileum while preserving the ileocecal region (18, 19). During the surgery, we found that the duplication deformity was located in the ileocecal region, 5 cm away from the ileocecal region. Although studies have shown a high risk of intestinal fistulas after multiple intestinal anastomoses (5), we used the above methods to completely remove the two cysts and perform intestinal reconstruction while successfully retaining the ileocecal part. No post-operative complications were observed. This demonstrates that the proposed method is safe and feasible.

Multiple duplications of the small intestine are extremely rare, the number of cases is small, there are no systematic reports or comparative studies, and there is a lack of relevant diagnostic and treatment experience for reference. For multiple small intestine duplications adjacent to the ileocecal junction, long-term follow-up is required to determine the risk of recurrence after ileocecal preservation and mucosal resection.

## 4. Conclusions

Multiple small intestine duplications are rare. It is necessary to perform color Doppler ultrasonography and abdominal CT before surgery, and laparoscopic exploration during surgery can further reduce the risk of missed diagnoses. Ileocecus-preserving laparoscopic surgery (cystectomy and intestinal repair) is also safe and feasible when multiple small intestinal duplications are located near the ileocecal junction, and the lesions are close to each other.

## Patient's perspective

Patient's parents: “Thank you for doctors’ skill”. The doctors succeeded in relieving our child of chronic abdominal pain. I can't believe that there are two deformities in our baby's abdominal cavity.”

## Data Availability

The raw data supporting the conclusions of this article will be made available by the authors, without undue reservation.

## References

[1] Ludwig K De Bartolo D Salerno A Ingravallo G Cazzato G Giacometti C Congenital anomalies of the tubular gastrointestinal tract. Pathologica. (2022) 114:40–54. 10.32074/1591-951X-553 35212315 PMC9040549

[2] Zouari M Bouthour H Abdallah RB Hlel Y Malek RB Gharbi Y Alimentary tract duplications in children: report of 16 years’ experience. Afr J Paediatr Surg. (2014) 11:330–3. 10.4103/0189-6725.143146 25323184

[3] Mayer J Bettolli M. Alimentary tract duplications in newborns and children: diagnostic aspects and the role of laparoscopic treatment. World J Gastroenterol. (2014) 20:14263–71. 10.3748/wjg.v20.i39.14263 25339813 PMC4202355

[4] Azzam A Abdulkarim AN Shehata A Mahran I Arafa A Arafat A A report of two infant cases operated for jejunal duplication cyst associated with malrotation and volvulus. Int J Surg Case Rep. (2020) 67:227–30. 10.1016/j.ijscr.2020.02.009 32113129 PMC7047138

[5] Grimm C Harter P Alesina PF Prader S Schneider S Ataseven B The impact of type and number of bowel resections on anastomotic leakage risk in advanced ovarian cancer surgery. Gynecol Oncol. (2017) 146:498–503. 10.1016/j.ygyno.2017.06.007 28610745

[6] Bower RJ Sieber WK Kiesewetter WB. Alimentary tract duplications in children. Ann Surg. (1978) 188(5):669–74. 10.1097/00000658-197811000-00015 718292 PMC1396776

[7] Yu M Fang Z Shen J Zhu X Wang D Shi Y. Double simultaneous intussusception caused by Meckel’s diverticulum and intestinal duplication in a child. J Int Med Res. (2018) 46:3427–34. 10.1177/0300060518777337 29968497 PMC6134666

[8] Karnak I Ocal T Senocak ME Tanyel FC Büyükpamukçu N. Alimentary tract duplications in children: report of 26 years’ experience. Turk J Pediatr. (2000) 42:118–25. 10.4103/0189-6725.143146 10936977

[9] Xiang L Lan J Chen B Li P Guo C. Clinical characteristics of gastrointestinal tract duplications in children: a single-institution series review. Medicine. (2019) 98:e17682. 10.1097/MD.0000000000017682 31689788 PMC6946480

[10] Martini C Pagano P Perrone G Bresciani P Dell’Abate P. Intestinal duplications: incidentally ileum duplication cyst in young female. BJR Case Rep. (2019) 5:20180077. 10.1259/bjrcr.20180077 31555466 PMC6750633

[11] Ren HX Duan LQ Wu XX Zhao BH Jin YY. Laparoscopic resection of gastric duplication cysts in newborns: a report of five cases. BMC Surg. (2017) 17:37. 10.1186/s12893-017-0234-x 28403863 PMC5388992

[12] Iwanaka T Hashizume K Kawarasaki H Tanaka K Kanamori Y Utsuki T Ileocecal resection in neonates and infants: a follow-up study. J Pediatr Surg. (1993) 28:110–2. 10.1016/s0022-3468(05)80367-7 8429463

[13] Malbert CH. The ileocolonic sphincter. Neurogastroenterol Motil. (2005) 17(Suppl 1):41–9. 10.1111/j.1365-2982.2005.00657.x 15836454

[14] Folaranmi S Rakoczy G Bruce J Humphrey G Bowen J Morabito A Ileocaecal valve: how important is it. Pediatr Surg Int. (2011) 27:613–5. 10.1007/s00383-010-2841-9 21243365

[15] Shafik AA Ahmed IA Shafik A Wahdan M Asaad S El Neizamy E. Ileocecal junction: anatomic, histologic, radiologic and endoscopic studies with special reference to its antireflux mechanism. Surg Radiol Anat. (2011) 33:249–56. 10.1007/s00276-010-0762-x 21184079

[16] Bhat NA Agarwala S Mitra DK Bhatnagar V. Duplications of the alimentary tract in children. Trop Gastroenterol. (2001) 22:33–5. PMID: . 11398245

[17] Tiryaki T Senel E Atayurt H. Anal canal duplication in children: a new technique. Pediatr Surg Int. (2006) 22:560–1. 10.1007/s00383-006-1654-3 16538439

[18] Catalano P Di Pace MR Caruso AM De Grazia E Cimador M. Ileocecal duplication cysts: is the loss of the valve always necessary. J Pediatr Surg. (2014) 49:1049–51. 10.1016/j.jpedsurg.2013.12.026 24888861

[19] Deguchi K Saka R Watanabe M Masahata K Nomura M Kamiyama M Ileocecal valve-sparing surgery for duplication cysts in the terminal ileum: two case reports and literature review. Surg Case Rep. (2022) 8:130. 10.1186/s40792-022-01483-w 35792950 PMC9259777

